# Tissue Level Mechanical Properties and Extracellular Matrix Investigation of the Bovine Jugular Venous Valve Tissue

**DOI:** 10.3390/bioengineering6020045

**Published:** 2019-05-14

**Authors:** Adam A. Benson, Hsiao-Ying Shadow Huang

**Affiliations:** Mechanical and Aerospace Engineering Department, Analytical Instrumentation Facility, North Carolina State University, R3158 Engineering Building 3, Campus Box 7910, 911 Oval Drive, Raleigh, NC 27695, USA; aabenson@ncsu.edu

**Keywords:** collagen crimp, elastin, microstructures, force-controlled mechanical testing

## Abstract

Jugular venous valve incompetence has no long-term remedy and symptoms of transient global amnesia and/or intracranial hypertension continue to discomfort patients. During this study, we interrogate the synergy of the collagen and elastin microstructure that compose the bi-layer extracellular matrix (ECM) of the jugular venous valve. In this study, we investigate the jugular venous valve and relate it to tissue-level mechanical properties, fibril orientation and fibril composition to improve fundamental knowledge of the jugular venous valves toward the development of bioprosthetic venous valve replacements. Steps include: (1) multi loading biaxial mechanical tests; (2) isolation of the elastin microstructure; (3) imaging of the elastin microstructure; and (4) imaging of the collagen microstructure, including an experimental analysis of crimp. Results from this study show that, during a 3:1 loading ratio (circumferential direction: 900 mN and radial direction: 300 mN), elastin may have the ability to contribute to the circumferential mechanical properties at low strains, for example, shifting the inflection point toward lower strains in comparison to other loading ratios. After isolating the elastin microstructure, light microscopy revealed that the overall elastin orients in the radial direction while forming a crosslinked mesh. Collagen fibers were found undulated, aligning in parallel with neighboring fibers and orienting in the circumferential direction with an interquartile range of −10.38° to 7.58° from the circumferential axis (n = 20). Collagen crimp wavelength and amplitude was found to be 38.46 ± 8.06 µm and 4.51 ± 1.65 µm, respectively (n = 87). Analyzing collagen crimp shows that crimp permits about 12% true strain circumferentially, while straightening of the overall fibers accounts for more. To the best of the authors’ knowledge, this is the first study of the jugular venous valve linking the composition and orientation of the ECM to its mechanical properties and this study will aid in forming a structure-based constitutive model.

## 1. Introduction

Venous valves are semi-lunar cusps that prevent retrograde blood flow in the venous system. For example, jugular vein valve insufficiency is a hypothesized etiology, given that many patients report Valsalva-associated maneuvers prior to a transient global amnesia event [1,2,3]. It is also hypothesized that increased intra-abdominal pressure is transmitted into the intracranial venous system, causing intracranial hypertension; jugular valve insufficiency may facilitate pressure transmission [4]. Moreover, chronic venous insufficiency (CVI) occurs when the saphenous venous valves in the vein are damaged or malfunctioning, leading to blood pooling at the distal end of the legs, causing swelling in the legs. Current treatments for CVI, such as venous valve replacement, have been developed and jugular venous valves were generally used in need of surgical replacement: replacing incompetent saphenous venous valves in the legs to prevent varicose veins, edema, poor circulation, valvular incompetence and all other symptoms of CVI. For example, Medtronic’s Contegra pulmonary valved conduit [5] and transcatheter Melody pulmonary valve [6] are based on glutaraldehyde-fixed trileaflet bovine jugular venous valves. Many current bioprosthetic replacements use fixated xenografts, since the better endotheliazed autologous vein segments take extreme surgical precision and are often unavailable [7]. However, fixated tissues tend to have warped mechanical properties and can affect valvular hemodynamic performance, which was shown in a study of the mitral valve [8]. Therefore, this study highlights the characteristics that should be desired when creating tissue-engineered substitutes, including mechanical properties, fibril orientation and fibril composition. 

Venous valves are arranged in different valvular patterns depending on the location throughout the venous system. The two most common valvular arrangements are tri-cuspid and bi-cuspid but uni-cuspid, quadri-cuspid and quinque-cuspid have been recorded [9,10]; however, uni-cuspid arrangements have been questioned as damaged bi-cupsid valves [9]. There are two enface sides of the venous valve—the parietal side and the luminal side. During the “opened position,” the parietal side faces the venous valve pocket and the luminal side faces away from the adhered venous wall. The parietal side is composed mostly of collagen and the luminal side has an overlaying elastic laminae, which is an extension of the venous wall [9]. Both sides are covered by an endothelium layer. Specifically, there are three types of venous valves: parietal valves, free parietal valves and ostial valves. Ostial valves are located at the entrance of a small vein into a larger one. Parietal valves are located at a junction where two veins of equal diameter merge into one vein. At these junctions, there is a valve present at each branch. Parietal valves are located distally to the heart of the bovine jugular vein, for example, where the maxillary and linguofacial veins converge. The third type, free parietal valves, are valves not at a junction [9]. 

Throughout different regions of the body, there are various types of collagen found in the extracellular matrix (ECM). Collagen fibers are made up of bundles of collagen fibrils and are usually found with an undulated pattern. The size and geometry of collagen fibers vary depending on the tissue. Different forms of collagen fibers include cord- or tape-shaped geometry [11]. Since collagen fibers give the extracellular matrix structural integrity, they are immensely relevant to this study. In the venous valve, the collagen microstructure is much thicker than the elastin microstructure and is located on the parietal side [9]. Previously, Huang and Lu [10,12] has used biochemical analysis of the venous valve tissue to investigate soluble collagen concentration and developed histological images using Masson’s trichrome stain. However, the study did not provide any implicit values regarding collagen crimp length, only a mere approximation. Knowing that crimp length is especially important because collagen fibers become increasingly stiffer when uncrimped. Elastin is defined by its ability to stretch up to 150% of its original length without attaining any permanent damage [13,14]. Physiologically, the luminal side is advantageous for the elastin location, because the luminal side experiences maximum stretch during anterograde flow. Elastin provides compliancy and recoil, allowing the tissue to undergo ongoing mechanical stress. Therefore, it is suggested that the elastin microstructure under tensile load has the ability to retract and aid the valve in closing during retrograde flow. 

Increased elastin has been observed in diseased venous valve leaflets [15], as has been collagen disorganization [16] and decreased collagen expression [17]. Given the increased pressure loads and concomitant tissue stresses in CVI, maladaptive venous valve tissue remodeling may parallel that of the pulmonary autograft in the Ross procedure [18,19] or that of the saphenous vein when utilized as a high pressure conduit in coronary bypass surgery [20]. Given how critical the elastin-rich ventricularis layer is to the function of heart valve leaflets, increases in venous valve leaflet elastin shown by Mouton et al. with progression of CVI may have important implications for the leaflet mechanical properties. Without knowing tissue-level mechanical properties and the ECM composition of healthy and diseased venous valve leaflets, it would be challenging to design replacement venous valves capable of long-term durability and normal physiologic function.

Previous mechanical tests of the venous valve included both a uniaxial mechanical test by Ackroyd et al. [21] and a biaxial test by Huang et al. [10,12,22]. The biaxial mechanical test better represents the physiological loading of the venous valve tissue and is, therefore, a better model of its mechanical properties. Huang and Lu [12] and Huang and Kaul [22] have previously reported the stress-strain curves, tangent moduli and constitutive models for the bovine jugular venous valve but the study was only limited to displacement-controlled loading condition. In this paper, the biaxial mechanical test applies three different force-controlled loading ratios, which allow the first attempt to characterize venous valve fiber rotation during loading. The mechanical properties could then be elucidated and compared to the investigated ECM’s collagen and elastin microstructures of the jugular venous valve.

The study by Saphir and Lev revealed images of longitudinal cross-sections of the jugular venous valve and Crissman provided the isolated elastin microstructure but to date, there is no study directly investigating the interplay of mechanical properties and the ECM in jugular venous valve tissue [23,24,25]. To the best of the authors’ knowledge, this paper is the first study to present microstructures that investigate the fiber orientation of both collagen and elastin, directly relating fiber orientation to jugular venous valves’ mechanical properties studied by a bi-axial mechanical test. To better understand the mechanical effects between elastin and collagen fibers, especially at the low-strain region, we incorporated sodium hydroxide digestion to identify elastin microstructure alone in the jugular venous valve. In this context, the mechanical properties and microstructure of the jugular venous valves is of interest. This paper is sought to outline the contribution of both the collagen and elastin microstructure to the physiological function of jugular venous valve tissue in hopes of redefining the properties that must be mimicked in bioprosthetic replacement.

## 2. Materials and Methods

**Figure 1:**
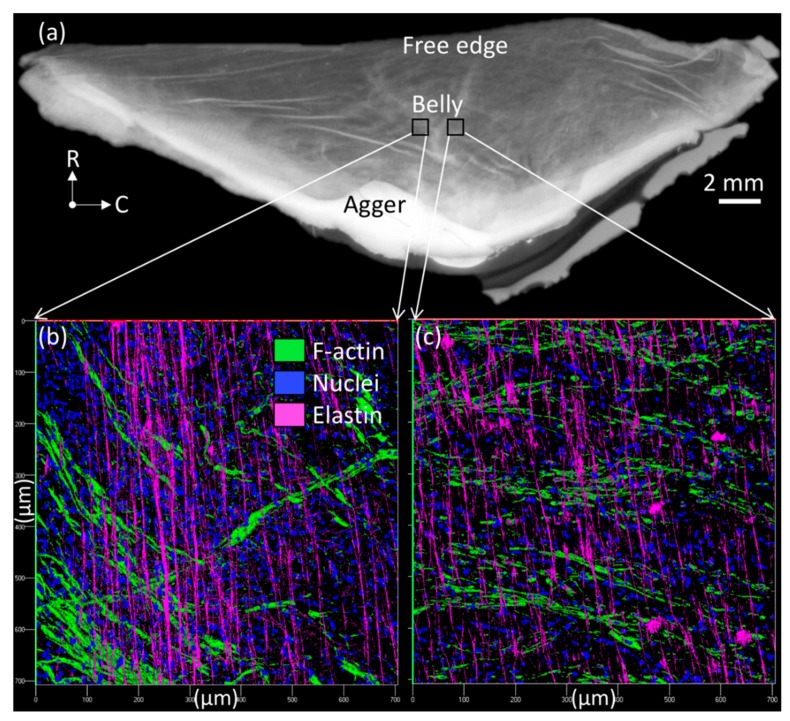
Jugular venous valve tissue viewed (**a**) from a regular high-resolution scanner and (**b**,**c**) in a LSM-710 confocal microscope (200×), where radial (R) and circumferential (C) directions were denoted in (a).

This study only focused on external jugular vein free parietal valves to keep all findings consistent. Additionally, only mature cows (Holstein breed, female, 10+ years old, ~1250 lbs weight) were used for the experiments since the age of the cow was shown to have noticeable effects on the collagen and elastin content [23]. Jugular venous valves were studied because their greater size aided mechanical testing and handling during NaOH (Fisher Chemical, Pittsburgh, PA, USA) treatments to isolate elastin. Jugular veins were removed from the mature cow and shipped to the lab overnight in a temperature-controlled environment. Therefore, the Institutional Animal Care and Use Committee (IACUC) review and approval is not required since tissues harvested from an animal that was euthanized for reasons other than our proposed study (culled). All connective tissue was cut off the exterior of the vessels. The veins were slowly turned inside out using forceps [12]. Venous valves were carefully dissected from the venous wall while submerged in Hank’s Balanced Salt Solution (HBSS). Since only free parietal valves were dissected, valves located at branches were not used for the experiments. After the dissection, venous valves were stored in HBSS at 1.11 °C (34 °F) until needed for further testing, which occurred no later than 72 h after dissection. A representative jugular venous valve tissue was showed in Figure 1, where a regular high-resolution scanner and a LSM-710 confocal microscope were used. Through a combination of z-stack and tile scanning, the three-dimensional cell nucleus distribution (by staining whole mount samples with DAPI (1:50 dilution) for 2 h and rinsing exhaustively with HBSS) and collagen fiber structures of the tissue across the entire surface area and through the thickness, were imaged based on collagen auto-fluorescence; the venous tissue was excited at 488 nm, with emissions collected from 490–590 nm.

### 2.1. Light Microscopy of the Collagen Microstructure 

Immediately following dissection, tissue was gently washed in HBSS. The sample was mounted on a microscope slide with HBSS and oriented so that the parietal side faced the eyepiece. The collagen microstructure was imaged with planar objectives on the inverted microscope (VWR Vista Vision, West Chester, PA, USA). The ImageJ (National Institutes of Health, MD, USA) ROI tool manager was used to quantify collagen orientation and crimp length and was related to the mechanical testing results. Specifically, the geometric analysis of the collagen crimp at relaxed conditions were associated with and used to investigate the circumferential direction’s inflection point on the stress-strain curve.

### 2.2. Isolation of the Elastin

Elastins are known to stretch to 150–200% local strain but it is the composition and architecture of the multi-component ECM which dictates the final outcome. In this study, we used NaOH digestion to identify elastin microstructure alone in the jugular venous valve. In brief, NaOH digestion—more frequently called hot alkali extraction—can degrade collagen due to its harsh conditions and it is recommended not to exceed 50–60 min at high temperatures [26,27]. The hot alkali extraction was made famous by Lowry and later modified by Lansing and has been a prevalent extraction method ever since. The method involves gelatinizing collagen and using weak alkali to remove other proteins from the elastin residue [27]. 

Dissected venous valve tissue was removed from HBSS and carefully placed into a glass beaker filled with 200 mL of 0.1 N NaOH solution heated to 75 °C by a LHS-720 Series Digital Hot Plate (Omega, Stamford, CT, USA). Temperature was monitored with an E4 Compact Thermal Imaging Camera (Flir, Wilsonville, OR, USA). The tissue was digested for 45 min following a published procedure of the aortic valve [28]. After the digestion procedure, the tissue was washed in HBSS and biochemically analyzed using a soluble collagen assay kit (Sircol; Accurate Chemical and Scientific Corp., Westbury, NY, USA). The sample’s wet weight mass and dry weight mass were measured via an analytical balance (VWR, West Chester, PA, USA). Next, the sample was vortexed in a 1-mL solution of acetic acid (0.5 M; Sigma-Aldrich, St. Louis, MO, USA) and pepsin (1 mg/mL Pepsin A (P-7000); Sigma Aldrich, St. Louis, MO, USA) in distilled water for 120 h. Collagen assay dye reagent of 0.1 mL was added to each sample and vortexed (VWR, West Chester, PA, USA) for binding. The sample was centrifuged with a Mini Spin (Eppendorf, Hamburg, Germany) at 13,400 RPM for 10 min. Excess dye and collagen extraction solution was removed, leaving the remaining solid mass of collagen content. One mL of 0.5 N NaOH was then added to the remaining collagen and vortexed. Solutions were placed in cuvettes and absorbance was measured by a Genesis 20 Spectrometer (Thermo Fisher Scientific, Waltham, MA, USA) at 550 nm [29,30]. Final collagen concentrations were determined by measuring the absorbance at 550 nm by a spectrometer versus collagen mass (both wet and dried) based on a collagen standard solution (Sircol Collagen Assay Collagen Standard 0.5 mg/mL). The proceeding process was repeated at 0.1 N NaOH 75 °C digestions for 45-min, 60-min and 75-min durations to determine an optimal duration for removing collagen content. 

The 75-min duration was deemed the best process for removing almost all collagen content in jugular venous valve tissue, despite the hot alkali method suggested not exceeding 50–60 min [26]. To established a better comparison of initial dry weight of the sample to the dry weight of the sample after digestion, fresh samples (n = 7) were lyophilized in a Free Zone 2.5 (Labconco, Kansas City, MO, USA) and weighed on a VWR analytical balance. After the 75-min heated NaOH digestion, the samples were gently washed in distilled water, lyophilized and weighed on an analytical balance again. Washing had to be very gentle because the remaining elastin substrate was delicate and sticky. Lastly, the process was followed with another Sircol soluble collagen assay to evaluate the remaining collagen concentration data.

### 2.3. Light Microscopy of the Elastin Microstructure

In this work, both light microscopy and scanning electron microcopy (SEM) were used to image the elastin microstructure. The 75-min 75 °C 0.1 N NaOH digestion was used on every sample before imaging. After digestion, the sample was gently washed in HBSS and moved to a microscope slide mounted with HBSS. An inverted microscope (VistaVision, VWR, West Chester, PA, USA) with planar objectives was used to image the elastin microstructure of the venous valve tissue. Removal of the collagen microstructure caused the elastin microstructure to unstretch. Image stitching of the same location at different focuses was used on some images since the corrugated samples had multiple focus depths.

### 2.4. Scanning Electron Microscopy of the Elastin Microstructure

Following the light microscopy, SEM was used to visually investigate damage yielded by the NaOH treatment used to isolate the elastin microstructure. For the purpose of comparison, undigested samples were also imaged. Samples were placed directly into a critical point drying holder for filters (samples had to be kept flat) and then into 3% glutaraldehyde in 0.1M NaPO4 buffer, pH 7.3 at 4 °C for one week. Samples were rinsed in three 30-min changes of cold 0.1 M NaPO4 buffer, pH 7.3 (by moving the holder from one jar to another), followed by 30-min changes in cold 30% and 50% ethanol (EtOH). The sample holder was moved to a jar of 70% EtOH and held overnight at 4 °C. Completed dehydration with a 30-min change of cold 95% EtOH was followed by a 60-min change to cold 100% EtOH, warming to room temperature and two 60-min changes of 100% EtOH. Samples were critical point dried with a Samdri-795 critical point dryer (Tousimis, Rockville, MD, USA) for ten min at critical point in liquid CO_2_. Samples were held in the desiccator overnight. All samples were sputter-coated (Hummer 6.2 Sputter System, Anatech USA, Union City, CA, USA); the non-digested samples were flat and coated with 25 Å from two sides plus 25 Å on top. The digested samples, which were convoluted, were coated with 25 Å from four sides plus 25 Å on top. Samples were returned to the desiccator until viewed. Samples were viewed in a JEOL JSM-5900LV SEM at 15 kV.

### 2.5. Force Control Mechanical Testing

Immediately following dissection, tissue was gently washed in HBSS. The mechanical test was conducted on a biaxial tester—the BioTester 5000 (CellScale, Waterloo, Ontario, Canada). Four rakes with five tungsten tines each were used for the boundary conditions of the venous valve tissue when testing [12,22,29,31]. The rakes were spaced in the radial and circumferential direction by 4,500 µm during resting conditions. During testing, the venous valve tissue was submerged in HBSS and heated to a constant 37 °C. During all testing, only the belly region of the venous valve tissue was tested, removing the excess tissue that overlaid the tungsten rakes. The tissue was mounted so that the radial direction of the belly region aligned on the y-axis and the circumferential direction aligned on the x-axis of the BioTester [12,22,29,31]. Before testing, a 10 mN preload was loaded in both the radial and circumferential directions. If this consistent preload was not applied, the samples could not be equally compared since they began at different initial strain. The following loading ratios were implemented into the mechanical test: (1) The 1:1 ratio was tested to 900 mN. The tissue was stretched with the same force in both the circumferential and radial directions; (2) The 1:3 ratio was loaded with 300 mN of force in the circumferential direction and 900 mN of force in the radial direction; and (3) In the 3:1 ratio, the circumferential direction was loaded with 900 mN and the radial direction was loaded with 300 mN. The extracted data were averaged across seven samples (n = 7) for each loading condition.

### 2.6. Statistical Analysis

Statistical analyses were conducted using JMP (SAS, Cary, NC, USA). One-way ANOVA was used to test the significance of data collected for the wavelength and amplitude of collagen crimps. Statistical significance was tested at *p* < 0.05 to determine if it was appropriate to use data to form a collagen crimp model for strain analysis.

## 3. Results and Discussion

### 3.1. Anatomical Findings during Dissection

Out of the 55 total bovine jugular veins (~30 cm long vein segments), there were 159 free parietal valves. One-hundred eleven valves (111) were bi-cuspid, 46 of the valves were tri-cuspid and 2 of the valves were quadri-cuspid. In addition, it was noticed that not entirely absent in venous valve leaflets is the glycosaminoglycan-rich spongiosa layer characteristic of semilunar heart valve leaflets. In the heart valve leaflets, the central spongiosa layer has been hypothesized to function as a mechanical dampener [28,32].

In the jugular vein, 28.93% of venous valves were tri-cuspid, whereas in the saphenous vein, all of the venous valves dissected are bi-cuspid [10]. The saphenous vein’s bi-cuspid geometry is always oriented so that its coaptation is colinear to its longitudinal direction of the venous wall’s elliptical cross section [9]. The superficial and deep veins in the legs always have elliptical cross sections due to the compressive force between the subcutaneous fascia and/or muscle [33]. Edwards concluded that the alignment is most likely mandatory for a tight closure of the venous valve [33]. In other words, if the compressive force was oriented perpendicular to the coaptation of the venous valve, the bi-cuspid valve would have an ineffective closure. Comparatively, the jugular venous valve has been found to both have a common occurrence of bicuspid and tricuspid valves. This is in agreement with a previous study which discovered that bovine proximal external jugular vein segments tend to be bi-cuspid and distal external jugular vein segments tend to be tricuspid [12]. The common appearance of tri-cuspid valves may be in response to the vein in vivo being shaped distally with circular cross sections as opposed to elliptical, as in the proximal external jugular vein and saphenous vein. The smaller loads on the exterior of the vessel are justified by smaller muscles located in the neck compared to the chest and leg. The lateral pectoral groove most likely applies more compression proximally to the venous walls than the jugular groove applies distally to the venous walls. The lateral pectoral groove is squeezed by both the brachiocephalicus and descending pectoral muscles [34]. Perhaps when veins are less loaded and have circular cross sections, tri-cuspid valves perform better and remain competent longer than bi-cuspid valves. This would explain the cuspid distinction between the venous valves located in the distal external jugular vein segments and the proximal external jugular vein segments and saphenous veins. 

Particularly, it is an interesting investigation because traditional percutaneous venous valve designs often have stents with circular cross sections but they are designed with bi-cuspid geometry [7,14]. Further providing justification, stent diameters are often oversized by 10% and the rigidity of the stent in some cases will dictate the shape of the host vein [7,14]. Due to the venous valves physiological existence, it may be true that the synergy of venous wall cross-sectional shape and cusp geometry play a role in overall venous valve competence. If the hypothesis is correct that tri-cuspid valves occur in the jugular vein due to the vessel’s segments with circular cross sections, it could be that tri-cuspid valves would outperform bi-cuspid valves in the circular cross-sectioned stents that are often used in the lower legs.

### 3.2. Light Microscopy of the Collagen Microstructure

**Figure 2:**
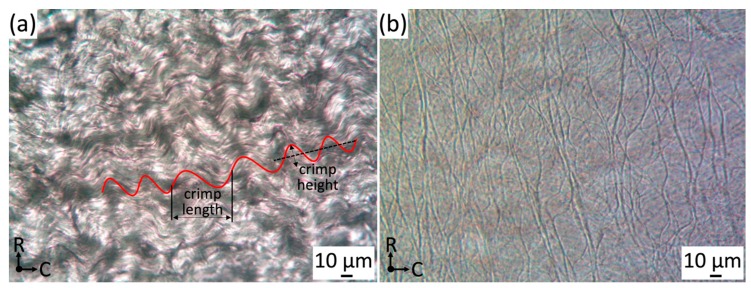
(**a**) Collagen crimp light microscopy images focused on the parietal side of jugular venous valve tissue (400×), where C = circumferential and R = radial directions. (**b**) Light microscopy of isolated elastin microstructure (luminal side) (400×). Please note that it was a projected image since several different focus levels were included in the image.

Collagen fibers are found in nearly all tissues and maintain their overall structure [35]. The collagen fiber network resides on the parietal side of the jugular venous valve just below the endothelium. Relaxed collagen fibers in the jugular venous valve have a wavy pattern and align in parallel with each other, orientating in the circumferential direction. An image of the collagen microstructure is shown in Figure 2a. Orientation of collagen fibers was measured through the center of the undulated collagen fiber segments (~150–200 µm length (Figure 2a)). It was found that most fibers align within a 10° range from the circumferential axis with an interquartile range of −10.38° to 7.58° (n = 20) and all fibers have an immediate parallel relation to neighboring fibers. The wavy pattern is a protection against direct tension on the collagen microstructure, similar to that of the aortic valve [36]. During loading, the collagen fibers must uncrimp before being loaded under tension. Since collagen ruptures at low strains (10–20%) and begins to yield at lower strains (1–2%), the crimps protect collagen fibers from overextending, undergoing plastic deformation and rupturing [37,38]. 

By analyzing the collagen fiber as a sinusoidal wave, we can make an approximation of how much strain it takes to uncrimp the collagen fibers. The average wavelength and amplitude was measured at 38.46 ± 8.06 µm and 4.51 ± 1.65 µm (n = 87), which was used to form the sinusoidal function. Therefore, the sinusoidal function was shown below:

(1) $$y=4.51\;\sin\left(\frac{2\;\pi }{38.46}x\right)$$

The distance of a linearly stretched sinusoidal wave can be calculated using the arc length of a curve formula shown in Equation (2).

(2) $$r=\;\int_{a}^{b}\sqrt{1+\;{\left(\frac{dx}{dy}\right)}^{2}}dx$$

(3) $$\int_{0\;µm}^{38.46\;µm}\sqrt{1+\;{\left(\left(\frac{2\;\pi }{38.46\;µm}\right)\left(4.51µm\right)\cos\left(\frac{2\;\pi }{38.46}x\right)\right)}^{2}}dx=43.24\;µm$$

After the elongated sinusoidal wave had been calculated, it was possible to find the percent true strain from uncrimping in the circumferential direction.

(4) $$\ln(\frac{43.24\;µm}{38.46\;µm})\;=\;11.71\%$$

### 3.3. Isolation of the Elastin Microstructure

To the best of the authors’ knowledge, this study is the first to use a variation of the hot alkali method to isolate the elastin microstructure in jugular venous valve tissue. The hot alkali method involves incubating a sample in a diluted NaOH solution, usually for 45 min, but some variations include extended time [39]. Remains of collagen were investigated with a Sircol Collagen Assay kit after the 0.1 N NaOH 75 °C heat treatment for the following timed tests: 45, 60 and 75 min. 45- and 60-min treatments still showed evidence of remaining collagen concentrations. The 75-min tests showed that in six out of eight samples, soluble collagen was completely removed from the venous valve’s substrate. The remaining two samples had very low collagen concentrations compared to previous collagen assays conducted on non-digested tissue (361–616 mg/g dry weight) and were determined viable for imaging of the elastin microstructure [12]. 

After digestion, the samples had negligible or no amounts of collagen and were very delicate with only 11.63 ± 2.64% ($\frac{digested\;dry\;weight}{non-digested\;dry\;weight}$) remaining. Comparisons of before and after digestion showed that the sample had noticeable amounts of shrinkage. It indicated that in relaxed conditions, the elastin microstructure was held in a preloaded position. In addition, the digested sample was very sticky, which was characteristic of purified tropoelastin [26]. However, the digestion was not perfect. Two out of the eight samples still had low amounts of soluble collagen concentrations after biochemical analysis. In defense of the digestion procedure, the remaining amounts of soluble collagen concentration, if any, were slim to none considering such high soluble collagen concentrations before digestion (proximal: 361 mg/g dry weight, middle: 439 mg/g dry weight and distal: 616 mg/g dry weight) [12].

### 3.4. Light Microscopy of the Elastin Microstructure

After isolating the elastin, the specimen was carefully transferred to a microscope slide, mounted with HBSS and imaged via light microscopy with a planar objective. Figure 2b showed the elastin microstructure located on the luminal side of the belly region of venous valve tissue and the large cusp fibers of the elastin microstructure having a radially crosslinked alignment was observed. We have observed that the light microscopy was difficult to focus because the remaining elastin microstructure had different regions of depth due to the sample shrinking and warping. Figure 2b needed to be stitched at several different focus levels to include the whole image. 

### 3.5. Scanning Electron Microscopy of the Elastin Microstructure

**Figure 3:**
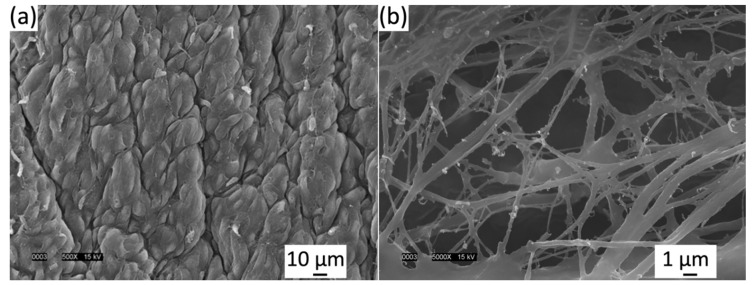
(**a**) Luminal side of venous valve tissue (**a**) before 75 °C NaOH digestion and (**b**) after 75 °C NaOH digestion viewed in a JEOL JSM-5900LV scanning electron microscope (SEM) at 15 kV. Elastin fibers are imaged clearly but damage can be seen.

Light microscopy was well-suited for qualitatively describing the macro scale of the elastin microstructure because of its fast preparation compared to SEM but for further magnification, SEM was advantageous for image quality. Therefore, SEM was used to test the quality of the isolated elastin digestion. Figure 3a showed the luminal side of the venous valve tissue before 75 °C NaOH digestion. Figure 3b showed the luminal side of venous valve tissue after 75 °C NaOH digestion. After ethanol dehydration and critical point drying, the samples were imaged in both non-digested and digested states. The non-digested samples clearly showed the endothelial cells’ long axis alignment in the radial direction of the cusp shown in Figure 3b. The digested SEM images showed that not all of the basal lamina was digested but enough of it was removed to view parts of the elastin microstructure located in the belly region. The elastin microstructure showed no significant orientation at 5000× (Figure 3b) and also showed that certain fibers were damaged during the hot alkali digestion. These damaged fibers were disconnected and appear untaut, differing from the rest of the elastin fibers.

As seen in Figure 3b and other studies, SEM showed that not all elastic fibers orient radially [24]. Light microscopy (400×) showed the overall macro-scaled radial crosslinked orientation (Figure 2b), whereas SEM (5000–10,000×) imaged smaller anastomosing elastic fibers orienting in all directions. These results must be approached cautiously, because it must be noted that the SEM critical point drying could alter the elastin’s state, unlike light microscopy which yields more accurate imaging. Moreover, using SEM, it was discovered that freeze drying can fracture and twist the sample, where critical point drying was shown to shrink the sample [24]. The light microscopy imaging was not as clear or magnified as SEM, although the elastin microstructure was thought to be closer to its fresh state since the drying methods are avoided. 

The 75-min hot alkali digestion used was effective enough for high magnification images of the elastin microstructure using lower magnification images using light microscopy and SEM. High SEM magnification showed that glycoproteins of the basal lamina were still intact, covering up parts of the elastin microstructure. Along with not completely isolating the elastin microstructure, the high magnification of SEM (10,000×) showed considerable amounts of damage to small anastomosing elastic fibers. Current methods used to isolate the elastin microstructure would not be advised for the future mechanical testing of the elastin microstructure, because the damaged elastin as well as the unwanted remains of collagen and the basal lamina would minimize the significance of the mechanical test’s results. For future mechanical testing of the elastin microstructure, a more in depth NaOH digestion procedure or a collagenase digestion may be less invasive [30,40] of the elastin and allow for adequate mechanical testing of the isolated elastin microstructure.

### 3.6. Biaxial Mechanical Testing 

**Figure 4:**
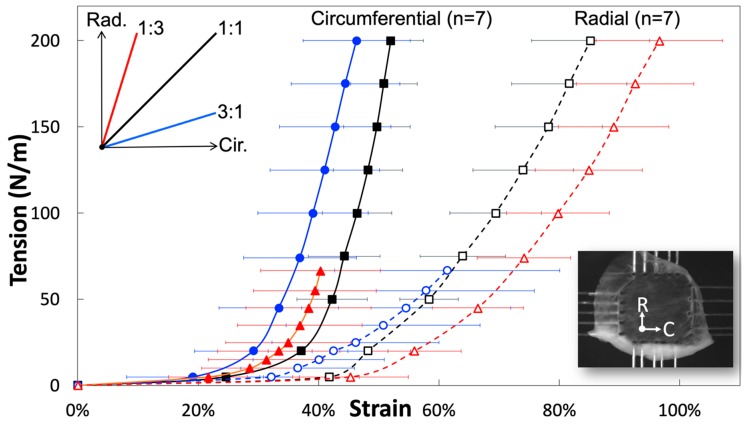
Representative circumferential and radial stress-strain curves (mean ± SEM) from fresh jugular venous valve leaflets, demonstrating the pronounced mechanical anisotropy of tissues and the effects of transverse loading (in-plane coupling).

A biaxial planar force-control mechanical test was used to investigate tissue-level fiber orientation and the rotation of the jugular venous valve. By implementing different loading ratios of circumferential: radial = 3:1, 1:1 and 1:3, the venous valve’s fiber orientation and rotation were elucidated from the extracted stress-strain curves (Figure 4) and the curves demonstrated the pronounced mechanical anisotropy of tissues and the effects of transverse loading (in-plane coupling). Stiffness values of the linear regions of the 1:1 ratio for the circumferential and radial directions were 29.34 ± 1.72 MPa and 11.38 ± 0.84 MPa respectively (black curves in Figure 4; Table 1). The linear region’s slope was evaluated between 75 N/m and 100 N/m of traction for both directions, using an average venous valve thickness, 40 µm. These values were similar to previous findings using a displacement-controlled mechanical test [12]. The averaged circumferential direction began to stiffen (enter the heal region) at 24.6% true strain and became linear approximately at 40%. The venous valve, which has anisotropic mechanical properties, had a radial direction considerably less stiff than the circumferential direction, not exiting the toe region until approximately 42% true strain. The 1:3 ratio (red curves in Figure 4) was loaded with 300 mN of force in the circumferential direction and 900 mN of force in the radial direction. In the circumferential direction, the heel region began at lower strain values than in the 1:1 ratio (~22% true strain). In the radial direction, the heel region began at greater strain values than the 1:1 ratio (~46% true strain). This created a leftward and rightward shift for the circumferential and radial stress strain curves compared to the 1:1 ratio. However, the stiffness values of the linear region were very comparable between 1:1 and 1:3 ratios. The linear stiffness values for the circumferential and radial direction were 33.15 ± 3.29 MPa and 11.16 ± 0.98 MPa, respectively (Table 1). In the 3:1 ratio, the circumferential direction was loaded with 900 mN and the radial direction was loaded with 300 mN (blue curves in Figure 4). The circumferential and radial directions both had leftward shifts on the stress strain graphs. The heel regions of both the circumferential and radial directions began stiffening before the 1:1 ratios corresponding directions (~19%, ~32%). The linear stiffness values for the circumferential and radial direction were 30.19 ± 2.78 MPa and 8.41 ± 1.54 MPa, respectively (Table 1).

Stiffness of the linear regions of all ratios closely resemble each other, except the radial 3:1 ratio could be due to rotated elastin fibers. These stiffness values showed that the linear region of the circumferential direction was close to three times stiffer than that of the radial direction in all loading ratios, except for the 3:1 ratio (due to its manipulated radial fiber components). The apparent differences between the ratios was the strain value at which the heel region begins. The 1:1 ratio showed that the jugular venous valve tissue’s mechanical properties were planar anisotropic. The circumferential properties were substantially stiffer and discovered to be caused by the strict collagen alignment in the circumferential direction. The 1:3 ratio showed a leftward and rightward shift of the circumferential and radial directions on the stress-strain curves, respectively. This was caused by the reaction of the ECM to the loading condition and Poisson’s ratio. Light microscopy images showed that the elastin fibers are crosslinked and oriented mostly radially (Figure 2b). During the 1:3 ratio loading, the elastin fibers straighten out in the radial direction and the circumferentially parallel-oriented collagen fibers remain oriented circumferentially. One significant difference in the loading ratios was overall stress; the 1:1 ratio has much more overall stress induced on the tissue than the 1:3 ratio, because both biaxial directions are loaded with 900 mN. In the 1:3 ratio, the circumferential direction was only loaded with 300 mN, while the radial direction was loaded with 900 mN. During 1:3 ratio testing, when the circumferential direction was at low strains, the radial direction was already at much higher strains. The overall stress of the radial direction being higher, while the circumferential direction was still loaded at low strains, made the stress-strain curve’s circumferential direction shift leftward (or the heel region stiffen at lower strains). When the radial direction was at maximum force, the circumferential direction’s strain was much less than in the 1:1 ratio. This results in the radial direction’s heel region appearing to be less stiff because of Poisson’s ratio. The overall difference of stress induced on the tissues from each loading ratio explained the leftward shift of the 1:3 ratio circumferential direction and the rightward shift of the 1:3 ratio’s radial direction when compared to the 1:1 ratio.

The 3:1 ratio did not follow what was expected if only Poisson’s ratio was considered. With higher forces in the circumferential direction and lower forces in the radial direction, the authors expected that the radial direction’s heel region would stiffen and the circumferential direction would appear less stiff. This would be true if the re-alignment of the ECM had not affected its mechanical properties during loading. The radial direction’s heel region stiffened at lower strains as expected but its linear region was not of the same stiffness as the other ratios and the circumferential direction’s heel region stiffened unexpectedly at lower strains. The re-alignment of the ECM can explain this stress-strain curve shift and the more compliant radial direction. In the circumferential direction, the elastin contributes to low strain properties. While the loading ratio deformed the sample orienting elastin fibers circumferentially, the heel region began to stiffen at lower strain values. This created a leftward shift of the 3:1 ratio. Since less elastin was aligned radially at higher strain values, the linear region of the radial direction appeared less stiff (8.41 ± 1.54 MPa). This was noticeably less stiff then the similar 1:1 and 1:3 radial directions’ linear region’s stiffness (11.38 and 11.16 ± 0.98 MPa). The linear region’s stiffness of the circumferential direction was unaffected by the elastin’s re-orientation because at higher loads, uncrimped collagen completely takes over its mechanical properties in the circumferential direction. The circumferential directions’ linear region’s stiffness for the 1:1, 3:1 and 1:3 ratio are 29.34 ± 1.72 MPa, 30.19 ± 2.78 MPa and 33.15 ± 3.29 MPa, respectively (Table 1). 

After all venous valve tissue was tested much past permanent slipping of collagen fibrils, mechanically tested close to rupture values. If collagen fibrils constantly slipped during loading, venous valves would be plastically deformed and soon destroyed. Despite these high loading values, the test was important in proving that the elastin layer contributes to both the circumferential and radial directions at lower strains. During the 3:1 loading ratio, the contribution of elastin in the circumferential direction was exaggerated and resulted in the stiffening of the curve at lower strain conditions. The force control test was also important for explaining that the collagen layer completely takes over the circumferential mechanical properties at higher loads when uncrimped. When elastin oriented partially circumferentially, the 3:1 ratio’s circumferential linear region did not experience stiffened values. Lastly, the test was important in showing that at higher loads the radial direction’s mechanical properties were still dominated by its elastin layer. With its elastin fibers oriented partially circumferentially, the 3:1 ratio’s radial linear region appeared less stiff. The circumferential direction’s inflection point of the 1:1 ratio occurs at ~24.6% true strain. It was hypothesized that straightening of the fibers accounts for the rest of the strain until the inflection point. Therefore, the authors hypothesized that straightening of the fibers account for ~12.89% of true strain. These results seem justified because collagen fibers did appear wavy in addition to the crimps. Additionally, the results seem physiologically similar to the aortic valve, where crimping accounts for 23% strain and straightening of the fibers accounts for 17% strain [38]. 

While collagen was uncrimping at low strains it was believed that the already pre-loaded elastin crosslinked mesh accounted for a majority of the circumferential mechanical properties. This assumption was reinforced by the force control testing. We believed elastin to be preloaded, because during the elastin isolation process the venous valve tissue consistently formed a corrugated radial direction and, when fixed, appeared stretched at the end of the digestion. Eventually, the strain reached the point where collagen was uncrimped. Once collagen was uncrimped, it immediately stiffened and the results of the stress-strain curve and elastin were no longer relevant in the stress-strain results in the circumferential direction. However, it was shown to still play a role in the radial direction’s linear region by the force-control data. This mechanical testing should be followed with an isolated elastin microstructure mechanical test of the jugular venous valve tissue. Before this test is possible, a method must be perfected to isolate the elastin microstructure without damaging it. A collagenase digestion [30,40] may be a better option than the heated NaOH digestion for future work.

The bi-layer anatomy of the venous valve allows the radial direction to be much more compliant than the circumferential direction. The parietal side has strict collagen alignment circumferentially and the luminal side has its large elastic fibers crosslinked orienting radially. Collagen orienting circumferentially across the venous valve may have the ability to guide the valve from the closed to open position, extending the compliant radial direction. In this case, individual collagen fibers running parallel causes the venous valve, when opening, to fold between collagen fibers. This fold is believed to be guided by the crypts. Crypts are located along the parietal side of the venous valve [9]. Similarly, in the aortic valve, collagen fibers stabilize motion during mid-systole [41]. The collagen fibers, during the venous valve’s closed position, are expected to carry the load of the above pressure column of blood. For example, cows spend the majority of their day (7–12 h) grazing with their head in the down position causing a pressure column from gravity [42]. In humans, pressure columns directed against the pockets of the jugular venous valve do not happen in daily activities due to our upright nature but do happen occasionally due to pressure wave impulses caused by coughing, straining or exterior compression of a venous segment [9]. In this case, the venous valve cannot completely prevent reflux but it will still partially mute it [9]. During pressure waves, venous valves are very important, because previous research has questioned if competent jugular venous valves obstruct reflux to the brain, preventing neurological disorders [43]. The strength of the venous valve tissue comes from collagen and causes it to have a breaking strength twice of the associated venous wall [21]. When loaded with reflux flow, collagen will uncrimp and mute the pressure wave. During the 1:1 loading ratio, the circumferential direction did not begin to acquire considerable force until the inflection point at 27.5% true strain. We hypothesize this to be the point at which collagen fibers are uncrimped and straightened. After the inflection point, the stiffness of the circumferential direction rapidly increased until the linear region, characteristic of yielding collagen fibers. 

## 4. Conclusions

Overall, the goal of this study was to conclusively identify the mechanical properties of the venous valve’s ECM with three different loading conditions. The mechanical test characterized fiber rotation and we related this information to the imaged collagen and elastin microstructures. The information from the imaged collagen and elastin microstructures, along with its mechanical properties, were used to understand how the bi-layer ECM contributes to the venous valves physiological function. In our study, light microscopy offers the first images of the venous valve’s isolated elastin microstructure unaffected by these drying methods. For the first time, venous valve tissue’s anisotropic behavior has been explained by the venous valve’s bi-layer ECM. A heated 0.1 N NaOH digestion isolated the elastin microstructure developing accurate light microscopy imaging of venous valve tissue without invasive drying procedures, such as with previous SEM. Additionally, light microscopy of the collagen microstructure allowed first time characterization of crimp effects on venous valve mechanical properties, indicating crimp allows about 12% strain. Collagen aligning circumferentially and elastin orienting radially, causes the circumferential direction to be stiffer and the radial direction to be more compliant. Force-control testing also provided proof that the elastin’s crosslinked mesh accounts for circumferential mechanical properties at low strains and affects the radial direction’s tangent modulus of elasticity at higher strains. This new knowledge of venous valve tissue-level microstructures is important for advances in basic venous physiology and, additionally, will be important for novel approaches for preventing venous valve incompetence. 

## Figures and Tables

**Table 1:** Modulus of elasticity of venous valvular tissue under three different loading ratios (mean ± SEM).

Modulus	1:1	1:3	3:1
Circumferential	29.34 ± 1.72 MPa	33.15 ± 3.29 MPa	30.19 ± 2.78 MPa
Radial	11.38 ± 0.84 MPa	11.16 ± 0.98 MPa	8.41 ± 1.54 MPa
